# XDecompo: Explainable Decomposition Approach in Convolutional Neural Networks for Tumour Image Classification

**DOI:** 10.3390/s22249875

**Published:** 2022-12-15

**Authors:** Asmaa Abbas, Mohamed Medhat Gaber, Mohammed M. Abdelsamea

**Affiliations:** 1 School of Computing and Digital Technology, Birmingham City University, Birmingham B4 7AP, UK; 2 Faculty of Computer Science and Engineering, Galala University, Suez 435611, Egypt; 3 Department of Computer Science, Faculty of Computers and Information, University of Assiut, Assiut 71515, Egypt

**Keywords:** explainable artificial intelligence, convolutional neural networks, medical images classification, unsupervised pre-training, data irregularities

## Abstract

Of the various tumour types, colorectal cancer and brain tumours are still considered among the most serious and deadly diseases in the world. Therefore, many researchers are interested in improving the accuracy and reliability of diagnostic medical machine learning models. In computer-aided diagnosis, self-supervised learning has been proven to be an effective solution when dealing with datasets with insufficient data annotations. However, medical image datasets often suffer from data irregularities, making the recognition task even more challenging. The class decomposition approach has provided a robust solution to such a challenging problem by simplifying the learning of class boundaries of a dataset. In this paper, we propose a robust self-supervised model, called *XDecompo*, to improve the transferability of features from the pretext task to the downstream task. *XDecompo* has been designed based on an affinity propagation-based class decomposition to effectively encourage learning of the class boundaries in the downstream task. *XDecompo* has an explainable component to highlight important pixels that contribute to classification and explain the effect of class decomposition on improving the speciality of extracted features. We also explore the generalisability of *XDecompo* in handling different medical datasets, such as histopathology for colorectal cancer and brain tumour images. The quantitative results demonstrate the robustness of *XDecompo* with high accuracy of 96.16% and 94.30% for CRC and brain tumour images, respectively. *XDecompo* has demonstrated its generalization capability and achieved high classification accuracy (both quantitatively and qualitatively) in different medical image datasets, compared with other models. Moreover, a post hoc explainable method has been used to validate the feature transferability, demonstrating highly accurate feature representations.

## 1. Introduction

In recent years, both colorectal cancer (CRC) and brain tumour are considered among the most dangerous types of cancer, affecting both men and women around the world [1,2,3,4]. Deep learning has shown great potential as a diagnostic tool in handling various complex problems that automate the feature engineering process of the medical image analysis pipeline [5,6]. Convolutional neural networks (CNNs) are among the most effective deep learning algorithms, where impressive achievements have been made in the field of medical imaging [7,8,9]. The effectiveness of CNNs comes from their ability to detect local features within an image in a hierarchical manner. More precisely, CNN’s low-level layers are designed to encode generic representations for most vision tasks, while the high-level layers can learn more complex features. There are two different scenarios for training a CNN model: full training and transitional training. Training a model from scratch requires the initialisation of the whole network from end-to-end. This strategy is a less common approach and requires a large amount of annotated training data that are not typically available in the field of medical imaging [10]. Rather, transitional learning refers to extracting the knowledge gained from a pre-trained network and applying it to another (un)related task, making it faster and more practical with small datasets [11]. Traditionally, transitional training (or as commonly known as transfer learning) can be achieved by one of the following scenarios [12,13,14]: (a) “shallow tuning”, which aims at modifying only the network’s classification layer to handle the domain-specific task [15]; (b) “deep tuning” refers to retraining the weights of the whole network in an end-to-end fashion [16]; or (c) “fine-tuning” mode, which starts to retrain weights of the last layer and gradually retraining weights of more layers until the desired performance is reached [17].

Although transfer learning can contribute to the problem of data scarcity, the feature transferability process might be affected when domain-specific images are different from generic images used in CNN’s initial training. Thanks to the huge availability of unlabelled data, many researchers designed self-supervised learning solutions to cope with the lack of labelled training data, especially in the medical imaging domain [18]. In self-supervised learning, a pretext training model that is learned from a large amount of unlabelled data is utilised to generate useful feature representations that can be used to solve a new task called the downstream task. In [19], we previously developed a self-supervised model, called Self-Supervised Super Sample Decomposition (*4S-DT*), where a large number of unlabelled chest X-ray images were used in the pretext training task. Then, a fine-tuning strategy based on an ImageNet pre-trained network was employed to achieve coarse transfer learning to detect samples of new classes in a small dataset with data irregularity problems. *4*S-DT could deal with datasets with irregularities in their distributions. However, the downstream recognition component has been designed in a semi-automated way based on a pre-defined parameter to identify the number of sub-classes. More importantly, the quality of the sub-classes can affect the transferability capability of the model based on the underlying statistical distribution of the data. Motivated by these issues, in this paper, we propose a new model, called (*XDecompo*), where class decomposition is guided by the affinity propagation (AP) method [20]. *XDecompo* has a more generalisation capability, compared to *4S-DT*, due to the non-parametric nature of its class decomposition. Specifically, AP has shown more stable clustering results over *k*-means with a lower mean squared error when applied to greyscale images, even when compared to the best out of 100 runs of *k*-means [20]. Motivated by this experimental observation, we hypothesised that clear class boundaries between pairs of sub-classes have a positive effect on the transferability of features in the downstream task. As a consequence, the explainability of the model can be enhanced. Thus, the contributions of this work can be summarised as follows:

- Propose a new model, *XDecompo* (the developed code is available at (https://github.com/Asmaa-AbbasHassan/XDecompo accessed on 14 November 2022)), using an affinity propagation-based class decomposition mechanism to robustly and automatically learn the class boundaries in the downstream tasks.
- Investigate the generalisation capability of *XDecompo* in coping with different medical image datasets.
- Demonstrate the effective performance of *XDecompo* in feature transferability.
- Validate the robustness of *XDecompo* using a post hoc explainable AI method for feature visualisation, compared to state-of-the-art related models.

The paper is organised as follows: Section 2 reviews the state-of-the-art approaches in medical image classification with an overview of explainable AI in medical imaging. Section 3 discusses the main components of the *XDecompo* model. Section 4 describes our quantitative and qualitative experiments on two different medical image datasets. Section 5 discusses and concludes our work.

## 2. Related Work

Deep convolutional neural networks (*DCNNs*) are widely used in several diagnostic applications. For example, in [21], a CNN was used to classify colonic polyp into two classes; they are normal and abnormal based on patch images and several data augmentation processes. The adapted CNN consisted of three convolutional layers with different kernel sizes, each one followed by max-pooling layer. In [22], a CNN model was used to classify digitised images of colorectal cancer cases as benign hyperplasia, intraepithelial neoplasia, and carcinoma. The proposed architecture consisted of two convolution layers, max-pooling and ReLUs layers, and the training was based on stochastic gradient descent. In [23], the authors modified different pre-trained networks by replacing the classification layer with a global average pooling for colonic polyps detection. Moreover, CNN was used as a feature extractor in many works. For instance, in [24], a comparative study between a transfer learning from VGG-16 pre-trained network and a CNN architecture as a feature extractor, with a support vector machine (SVM) as a classifier to classify a large number of images patches into three histological categories; healthy tissue, adenocarcinoma, or tubulovillous adenoma. The results showed that the transfer learning techniques from a pre-trained network outperform the other method in terms of accuracy and time consumption. Similar to colorectal cancer, brain tumour is a common disease between adults and the elderly that severely affects the function of the body. There are numerous types of brain tumour, some benign, while others malignant. The most frequent types are glioma, meningioma, and pituitary tumours. Thus, early tumour detection plays a significant role in improving treatment outcomes and increasing patient survival. In [25], three dense layers with softmax activation function were combined at the inception of ResNet v2 to classify the brain tumour into three classes. In [26], transfer learning based on GoogleNet pre-trained network was used to extract features from brain MRI images and fed them into SVM and KNN as classifiers, separately, to classify the input image into three classes; glioma, meningioma, and pituitary tumours. In [27], a CNN architecture has been evaluated based on various pre-processing techniques. The architecture consisted of 18 layers divided into four convolution layers, each one followed by batch normalisation, Relu, max-pooling layer, and fully connected layer with softmax activation function to classify brain cancer into three types of tumour. In [28], a differential deep CNN model was proposed to classify MRI images into two classes (normal and abnormal). In [29], the authors used different sizes of the brain tumour region with several processing techniques of data augmentation to evaluate the performance of CNN based on the VGG-19 pre-trained network.

Although the transfer knowledge from a pre-trained network has superior outcomes, it sometimes fails when a dataset has data irregularity problems or imbalance classes [30,31,32]. Decomposition mechanisms can handle this issue by detecting the boundaries between classes and learning the local patterns of a dataset [33]. The basic idea of the decomposition mechanism is to break down the original classes in a dataset into simpler sub-classes. Then, each sub-class is given a new label associated with its original class and treated as an individual new class. Then, after training, those sub-classes are recollected again to compute the error correction of the final prediction. We argue that this can improve the classification performance of a dataset with irregularity distribution classes, which was clear from the experimental results in [34] that class decomposition was used in medical image classification for transfer learning, in a method called the Decompose, Transfer, and Compose (DeTraC) approach. In [35], self-supervised learning is used with several deep convolution neural networks for many image and video analysis applications. For example, in [36], a self-supervised learning approach based on context distortion was used in different problems in medical imaging; such as segmentation, classification, and localisation. In classification tasks, the method achieved classification progress when used to detect scan planes in 2D fetal ultrasound images. In [19], a self-supervised model based on sample decomposition (called *4S-DT* model) was proposed to capture complex features with highly nonlinear mapping between input and output data using stacked autoencoder (SAE) and density-based spatial clustering of applications with the noise (DBSCAN) method in the pretext task. *4S-DT* model has demonstrated its capability in coping with both data scarcity and irregularity problems.

## 3. Explainability AI in Medical Imaging

Although machine learning and deep learning models have achieved impressive prediction results, it is not clear what the model has learnt from the input data, leading to its decisions; these models are called black boxes. The explainability AI methods [37,38,39] give solutions to break down these black boxes and provide evidence to give confidence in the results in a way that experts in the field can understand. Different applications have widely depended on transparent techniques to introduce the results, such as face recognition [40], medical imaging [41,42], text translation [43], speech understanding, and generate human responses [44]. These techniques are important in healthcare to provide the community with models that are more trustworthy to match human experience. This is done by having the ability to indicate the significant pixels that drive the model to make its decisions and decide which class the patient belongs to (e.g., healthy or unhealthy) [45,46]. The visualisation of an explainable AI can be accomplished through various attention mechanisms such as: trainable, post hoc, soft, and hard attention scenarios [47,48]. A trainable attention mechanism is trained while the model is in the learning process to help the network focus on the important pixels of the image, while the post hoc attention method is applied after completing the training process with fixed weights to create a heatmap of occlusion [37], saliency [49], CAM [50], or Grad-CAM [51] maps. On the other hand, soft attention can be trained with the standard backpropagation method that is described by continuous variables, while hard attention is depicted by discrete variables; hence, it is non-differentiable and uses the crop method to focus on specific areas in the image. In this work, we adopted the Grad-CAM algorithm as a post hoc explainable method on top of the *XDecompo* model to quantify the contribution of each pixel of input images in the final prediction of the model and to explain the robustness of the feature speciality and transferability.

### XDecompo Model

**Figure 1:**
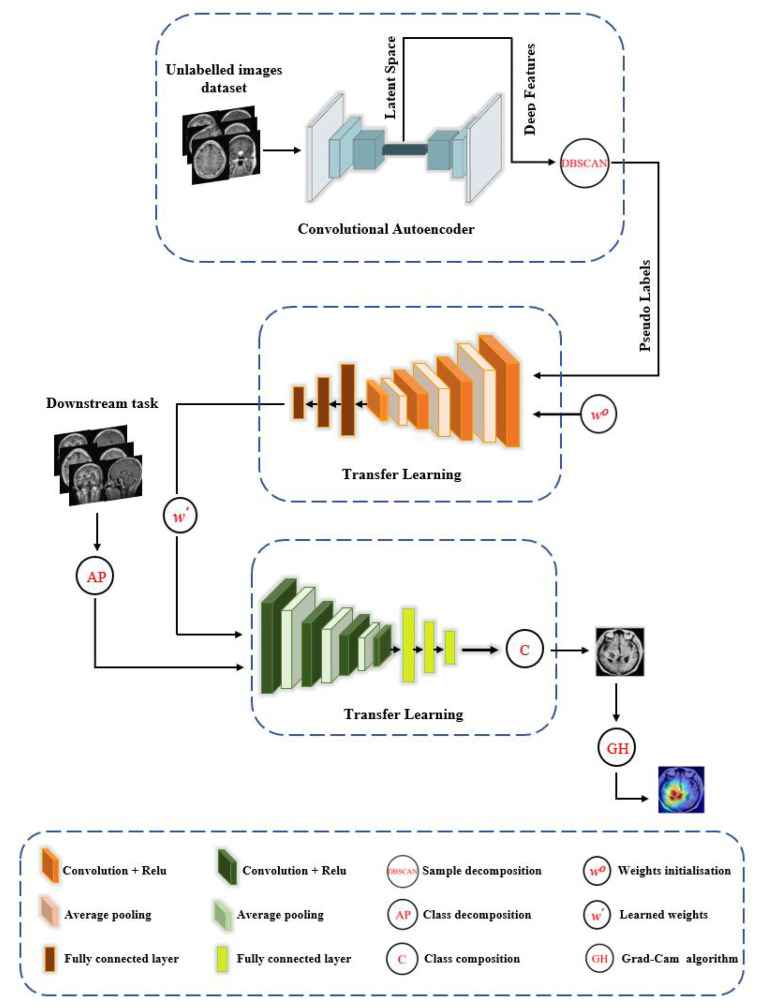
The framework of the *XDecompo* model.

*XDecompo* model is composed of the following four main stages, see Figure 1:

1. **Pseudo-Labels:** Extraction of deep local features from a huge number of unlabelled images using convolutional neural networks with a sample decomposition approach. We used the density-based spatial clustering of applications with the noise (DBSCAN) method [52] as a clustering algorithm for the annotation of pseudo-labels. DBSCAN is an unsupervised clustering algorithm that can cluster any type of data containing noise and outliers, without prior knowledge of the number of clusters.
2. **Pretext Training:** Using an ImageNet pre-trained network, such as ResNet-50, to classify pseudo-labelled images and achieve coarse transfer learning; where all layers were learnt as a deep-tuning mode to construct the feature space. The CNN model was trained with a cross-entropy loss function based on a mini-batch of stochastic gradient descent (mSGD) [53] to optimise the model during training.
3. **Downstream Training:** Utilisation of learned convolutional features with a novel class decomposition approach to solve a new task (i.e., downstream training) in a small dataset.
4. **Feature Visualisation:** Explanation and demonstration of the speciality of features learned/transferred by *XDecompo*, we used the Grad-CAM algorithm [51] as one of the most efficient interpretation techniques for computer vision tasks. It estimates the location of particular patterns in the input image, which guides the prediction of the *XDecompo* model, and highlights the patterns through an activation heatmap [54]. Grad-CAM is a generalisation of CAM that does not require a particular CNN architecture, contrary to CAM which requires an architecture that applies global average pooling to the final convolutional feature maps.

More precisely, we (a) use the convolutional autoencoder (CAE) [55] for the feature extraction in the pretext task; and (b) adopt the Affinity propagation (*AP*) [20] clustering method for the downstream task. CAE works by applying squared convolutional filter scans over the whole image to extract local features by compressing the image into a low dimension (e.g., encoder process), and then reconstructing the original values back (e.g., decoder process). The encoder process begins with several blocks, and each block consists of a convolution layer with a nonlinear activation function such as ReLU, and a pooling layer that downsamples the input image. The 2D convolution operation can be defined as:

(1) $$A\left(i,j\right)=\sum_{u=-2f-1}^{2f+1}\sum_{v=-2f-1}^{2f+1}x\left(i-u,j-v\right)w\left(u,v\right)+b_{ij},$$

where $A\left(i,j\right)$ is the output activation map in position (*i*,*j*), *x* is the input image, and *w* is the weights of square convolution filter with dimension (2f+1,2f+1). For *d* depth, the generated activation maps are the encoding of the input *x* in a low-dimensional space, which can be defined as:

(2) $$A^{d}=\sigma \left(x\times W^{d}+b^{d}\right),$$

where σ is an activation function, $b^{d}$ is the bias for d-th activation maps. Since our aim is to reconstruct the input *x* from the generated activation maps, we need to up-sample the compressed image. The reconstructed image $\hat{x}$ is obtained by:

(3) $$\hat{x}=\sigma \left(\sum_{d\in H}{\hat{A}}^{d}\times {\hat{W}}^{d}+b\right),$$

where $\hat{W}$ defines the inversion process on both dimensions of the weights and *H* is a group of activation maps. The cost function to minimise the error between $\hat{x}$ and *x* with the mean squared error (MSE) is defined as:

(4) $$MSE\left(\theta \right)=\frac{1}{2n}\sum_{i=1}^{n}{\left(x_{i}-\hat{x_{i}}\right)}^{2}$$

Once the training of the *CAE* is accomplished, *DBSCAN* was used to generate the pseudo-labels. Let an image $x^{i}$ (encoded as $h_{i}$) be density-connected to an image $x^{j}$ (encoded as $h_{j}$) with respect to Eps (the neighbourhood radius) and MinPts (the minimum number of objects within the neighbourhood radius of the core object) if there exists a core object $x^{k}$ such that both $x^{i}$ and $x^{j}$ are directly density-reachable from $x^{k}$ with respect to Eps and MinPts. Moreover, an image $x^{i}$ is directly density-reachable from an image $x^{j}$ if $x^{i}$ is located within the Eps-neighbourhood of $N_{Eps}\left(x^{j}\right)$, and $x^{j}$ is a core object. The Eps-neighbourhood can be defined as:

(5) $$\begin{array}{c} N_{Eps}\left(x_{j}\right)=\left\{x_{i}\in X|dis\left(x_{i},x_{j}\right)\leq Eps\right\}. \end{array}$$

*DBSCAN* results in *c* clusters, where each cluster is generated by maximising the density reachability relationship among images. The *c* cluster labels will be assigned to the $n^{\prime }$ unlabelled images (and will be presented as pseudo-labels) for the pretext training task of the self-supervision mechanism. The pseudo-labelled image dataset from the pretext task can be defined as $X^{\prime }=\left\{\left(x^{i},y^{c}\right)|c\in C\right\}$.

For the downstream task, the distribution of the image data *X* is broken down using the *AP* method into some classes *c* based on the extracted features $A^{d}$. The *AP* algorithm is an unsupervised clustering algorithm that does not require an initial number of clusters, and it depends on the idea of how messages are passed between data points; for example, how well the j-th point is appropriated to become an exemplar for the i-th point. Let the image dataset $X=x_{1},x_{2},...x_{n}$, and the function sE represents the similarity of two points (i,j) that can be described as a negative squared Euclidean distance as below:

(6) $$sE\left(i,j\right)=-{∥x_{i}-x_{j}∥}^{2},$$

where $x_{i}$ and $x_{j}$ are the positions of data points *i* and *j* in 2D space. The algorithm proceeds by alternating between two message-passing steps, which update two matrices as below:

(7) $$\rho \left(i,k\right)=sE\left(i,k\right)-max\left\{\alpha \left(i,k^{\prime }\right)+sE\left(i,k^{\prime }\right)\forall k^{\prime }\neq k\right\},$$

(8) $$a\left(i,k\right)=min\left(0,\rho \left(k,k\right)+\sum_{i^{\prime }\notin \left\{i,k\right\}}max\left(0,\rho (i^{\prime },k)\right)\right)i\neq k,$$

where ρ(i,k) refers to the “responsibility” matrix that quantifies how suitable $x_{k}$ is to serve as the exemplar for $x_{i}$ in comparison to other potential exemplars for $x_{i}$, and a(i,k) is the “availability” matrix that depicts how “proper” it would be for $x_{i}$ to choose $x_{k}$ as its exemplar, taking other points’ preferences for $x_{k}$ as an exemplar into account.

Here, in this work, we used the cosine similarity measure for *AP* to learn the boundary between certain features within each class. Cosine similarity is a structural similarity measure based on the idea that two vectors $\left(X_{i},X_{j}\right)$ are supposed to be similar if they have many neighbours in common, where a similarity of 0 indicates that the vector orientation is completely different, while a similarity of 1 indicates that the vector orientation is the same.

(9) $$D_{cosine}\left(i,j\right)=\frac{\left|X_{i}\cap X_{j}\right|}{\sqrt{\left|X_{i}|\right|X_{j}|}}.$$

As a fine transfer learning stage, *XDecompo* employs a fine-tune mode in the pre-trained model that has already been trained in the pretext stage to address the new downstream task (of the small annotated samples). Additionally, a class decomposition layer was adapted in *XDecompo* to split each class within the downstream dataset (denoted as dataset A) into *c* sub-classes, resulting in a new dataset (denoted as dataset B). Then, the new sets are given new labels, where each subset is treated as an individual class. Another important step is to reduce the feature space of a high dimension to a lower dimension using a principal component analysis (PCA) process [56], which is important for class decomposition to produce more homogeneous classes, reduce memory requirements, and improve framework efficiency. The class decomposition process can be defined as

(10) A=(A|L)↦B=(B|C)

where the number of examples in both *A* and *B* are equivalent, while C encodes the new generated labels associated with the sub-classes (e.g., $C=\{l_{11},l_{12},\cdots ,l_{1c},l_{21},l_{22},\cdots ,l_{2c},\cdots l_{nc}\}$). These sub-classes are then integrated back into the original classes in the dataset (the composition stage) and the final prediction is calculated accordingly.

Finally, we used the Grad-CAM algorithm to locate specific patterns in the input image, which informs the XDecompo model’s prediction, and emphasises the patterns using an activation heatmap. The basic idea behind Grad-Cam is that the weights of a convolutional layer are calculated using a gradient of the classification score $\partial x^{c}$ of a particular class *c*, for the feature activation map $\partial A^{d}$ on the *d*-th feature map. Then, the importance of the associated neuron of each feature map *d* is calculated by taking the global average pool of gradients at position (i,j) as follows:

(11) $$\varphi_{d}^{c}=\frac{1}{m}\sum_{i}\sum_{j}\frac{\partial x^{{}^{c}}}{\partial A_{ij}^{d}},$$

where *m* is the number of pixels in $A^{d}$. Finally, the sum of the product of the $\varphi_{d}^{c}$ with the corresponding feature map is performed under the ReLU activation function to obtain the final Grad-CAM heatmap as follows:

(12) $$H_{Grad-CAM}^{c}=ReLU\left(\sum_{d}\varphi_{d}^{c}A^{d}\right)$$

## 4. Experimental Setup and Results

This section discusses the experimental results, as well as the datasets used to validate the generality and explainability of XDecompo.

### 4.1. Datasets Description

In our work, we used two different medical image datasets, they are colorectal cancer histology dataset and brain tumour dataset (see below). These datasets have been selected due to (a) the presence of several data irregularity problems, including class overlap in terms of the morphological structure of objects within the images and the class imbalance problem; and (b) the huge availability of unlabelled related images [57].

**Figure 2:**
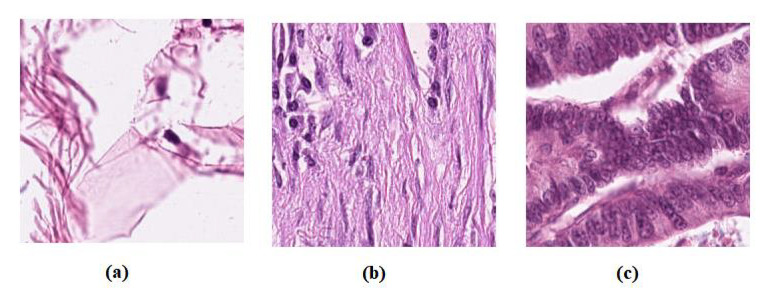
Example images from colorectal cancer test set: (**a**) ADI, (**b**) STR, and (**c**) TUM.

**Figure 3:**
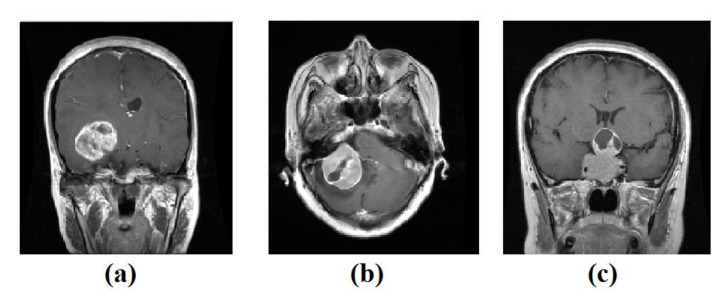
Example images from Brain tumour test set: (**a**) glioma, (**b**) meningioma, (**c**) pituitary tumour.

- **Colorectal cancer images dataset**, the labelled and unlabelled datasets were used from [58]; from the NCT Biobank, (National Center for Tumor Diseases, Heidelberg, Germany), and the UMM pathology archive (University Medical Center Mannheim, Mannheim, Germany). The dataset “NCT-CRC-HE-100K” [58] was used as unlabelled samples. With a total of 100,000 samples of (CRC) and normal tissue, all images are 224 × 224 pixels at 0.5 microns per pixel, the dataset divides into nine classes: Adipose tissue, background, debris, tumour epithelium, smooth muscle, normal colon mucosa, cancer-associated stroma, mucus, and lymphocytes.
- The dataset “CRC-VAL-HE-7K” [58] was used as a labelled dataset, a set of 7180 image patches divided into nine unbalanced classes, and all images are 224 × 224 pixels at 0.5 microns per pixel. In our experiment, we only used three classes, Adipose (ADI), stroma (STR) and tumour epithelium (TUM), which contain 1338, 421, and 1233, respectively. Then, the dataseet was divided into three groups: 60% for training, 20% for validation, and 20% for testing, see Table 1. Figure 2 shows example images from the test set. Note that there is no overlap with the cases in the unlabelled images, NCT-CRC-HE-100K.
- **Brain tumour images dataset**, we have used a public brain tumour dataset as unlabelled samples that contains a total of 253 images and divided into two classes: 155 tumours and 98 without tumours. The dataset is available for download at: (https://www.kaggle.com/datasets/navoneel/brain-mri-images-for-brain-tumor-detection access on 14 November 2022). We applied many data-augmentation techniques to generate more samples in each class, such as reflection, shifting, wrapping, and rotation with various angles. This process resulted in 45,960 brain tumour images.
- For the labelled dataset, we have used a set of 3064 brain tumours from Nanfang and General Hospitals, Tianjin Medical University, China: 1426 glioma, 708 meningiomas, and 930 pituitary tumour, available from [59], all images with size 400 × 400 pixels. The training set was randomly divided into 60% to fit the model, 20% for validation, and 20% as a test set, see Table 2. Figure 3 shows examples of images from the test set.

**Table 1:** The distribution of Colorectal cancer dataset.

Class Name	Training	Validate	Test	Total
ADI	856	214	268	1338
STR	270	67	84	421
TUM	789	197	247	1233

**Table 2:** The distribution of Brain tumour dataset.

Class Name	Training	Validation	Test	Total
glioma	855	285	286	1426
meningioma	424	141	143	708
pituitary_tumour	558	186	186	930

To quantitatively and qualitatively evaluate the performance of *XDecompo*, we utilised ResNet-50 ImageNet [60] as a backbone network. We used a fine-tuning mode by gradually training more layers and tuning the learning parameters until a significant high performance is achieved. Here, we consider freezing the lower layers and updating only the weights of upper layers (i.e., the last four layers), see Table 3. All experiments have been implemented in MATLAB 2021a on a desktop machine with an Intel(R) Core(TM) i3-6100 Duo processor @ 3.70 GHz, NVIDIA Quadra P5000GPU, and with a RAM capacity of 16.00 GB.

### 4.2. Self-Supervised Training on Unlabelled Images

**Figure 4:**
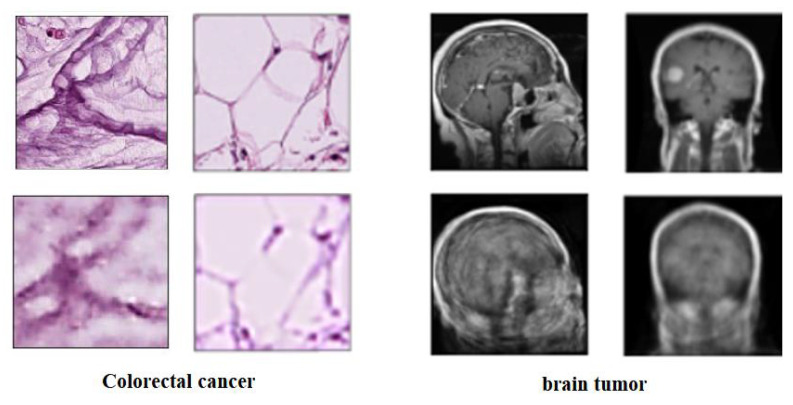
The SAE for unlabelled images; first row: the original images of the dataset, second row: the reconstructed images.

For the *4S-DT*, we used the SAE model with 600 neurons in the first hidden layer, 400 neurons in the second hidden layer, and 200 neurons for the latent space representation to train a randomly selected 50,000 unlabelled CRC images. In addition, the same SAE architecture was used for the 45, 960 unlabelled brain tumour images, see Figure 4.

**Figure 5:**
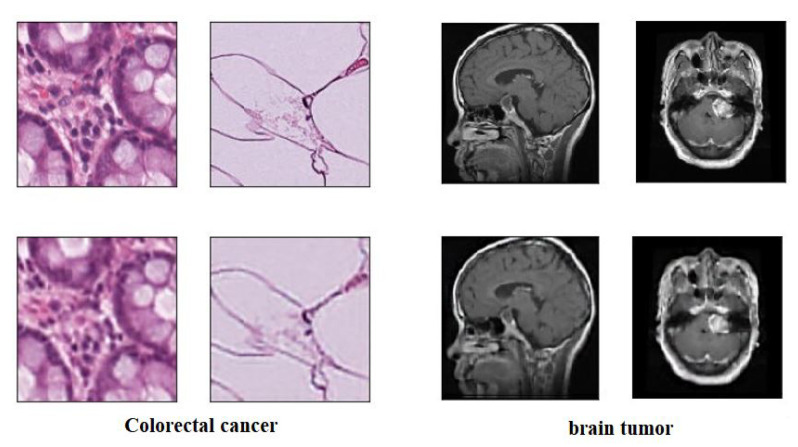
The CAE for unlabelled images; first row: the original images of the dataset, second row: the reconstructed images.

For *XDecompo*, we applied CAE on the same selected unlabelled images dataset. Here, we used two convolutional layers with a kernel size of 3 pixels and ReLU as an activation function. For the histological dataset, the number of filters in the first layer was set at a value of 64, and the second layer at a value of 32. Regarding the brain tumour image dataset, the number of filters in the first and second layers was set at a value of 32 and 16, respectively; see Figure 5.

Then, the extracted features obtained by the latent representation were used to construct the clusters (and hence generate the pseudo-labels) using the DBSCAN clustering algorithm. The *k*-nearest neighbour (*k*-NN) [61] was used to choose the ideal value (Eps). This is done by calculating the average of the distances of every point in the input data to its neighbours, corresponding to MinPts. The optimal value (Eps) and pseudo-labels for SAE and CAE are summarised in Table 4. The autoencoder (AE) models were trained with a 0.001 learning rate, with a mini-batch size of 128 and a minimum of 100 epochs. Finally, we used the ResNet-50 pre-trained *CNN* model for the coarse transfer learning stage (of the pretext task). The model was trained with a learning rate of 0.0001 and a drop learning rate of 0.9 every 10 epochs.

### 4.3. Downstream Class-Decomposition of 4S-DT and XDecompo

**Figure 6:**
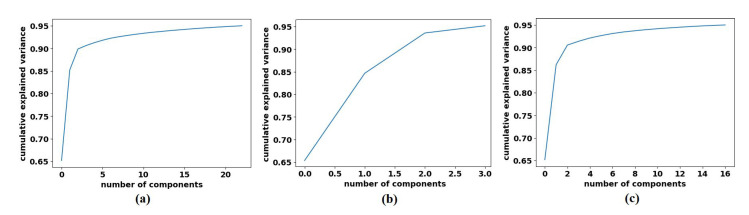
The explained variance of the principal components for each class in the CRC dataset: (**a**) ADI, (**b**) STR, and (**c**) TUM.

**Figure 7:**
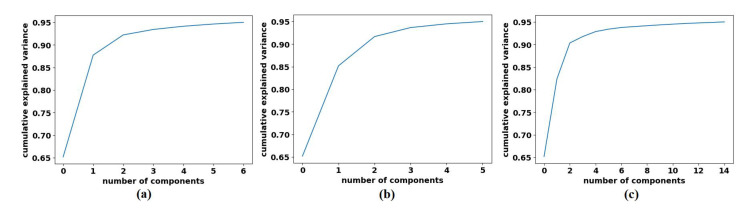
The explained variance of the principal components for each class in the Brain tumour dataset: (**a**) glioma, (**b**) meningioma, (**c**) pituitary tumour.

To decompose the labelled samples of the downstream dataset, we applied AlexNet [62] pre-trained network-based with a shallow-tuning mode to extract discriminative features from images of the original classes on each dataset. The learning rate was set at 0.0001, which was gradually reduced by 0.9 after every 3 epochs, the batch size of the training set was set at 128 with a minimum of 100 epochs, and the weight decay was set at 0.0001 to avoid overfitting and 0.9 for the momentum speed. The gradient descent (SGD) method was adopted to minimise the loss function. At this point, 1000 attributes had been collected; therefore, we utilised Principal Component Analysis (PCA) to make the feature space less dimensional. For the CRC dataset, we obtained 23, 3, and 16 for adipose, stroma, and tumour epithelium, respectively; see Figure 6. Regarding the brain tumour dataset, we obtained 6, 5, and 14 features for glioma, meningioma, and pituitary_tumour respectively, see Figure 7. For the class decomposition process, we used *k*-means algorithm [63]. *k*-means is one of the most popular unsupervised machine learning algorithms that divides a given dataset into a fixed number of clusters. The number of clusters is represented by a predetermined parameter *k*. The clusters are then placed as points, and all observations or data points are connected to the closest cluster that has similar properties. The procedure is then repeated with the new adjustments until the desired result is achieved (no or limited changes in cluster assignment). For *4S-DT* model, we used *k* = 2 (where the choice was based on the high performance obtained by the models *DeTraC* and *4S-DT* as indicated in [19,34]), and therefore, each class in (L) is divided into two sub-classes, each was assigned as a new label to the new dataset.

On the other hand, in *XDecompo*, the AP algorithm was used with the measure of cosine similarity. The damping factor value was set at 0.9 and 0.85 for the CRC and brain tumour dataset, respectively, with a value of 1000 for the maximum iteration and 50 for the convergence iteration parameter. The results of this process are reported in Table 5 and Table 6.

### 4.4. Classification Performance on CRC Dataset

**Figure 8:**
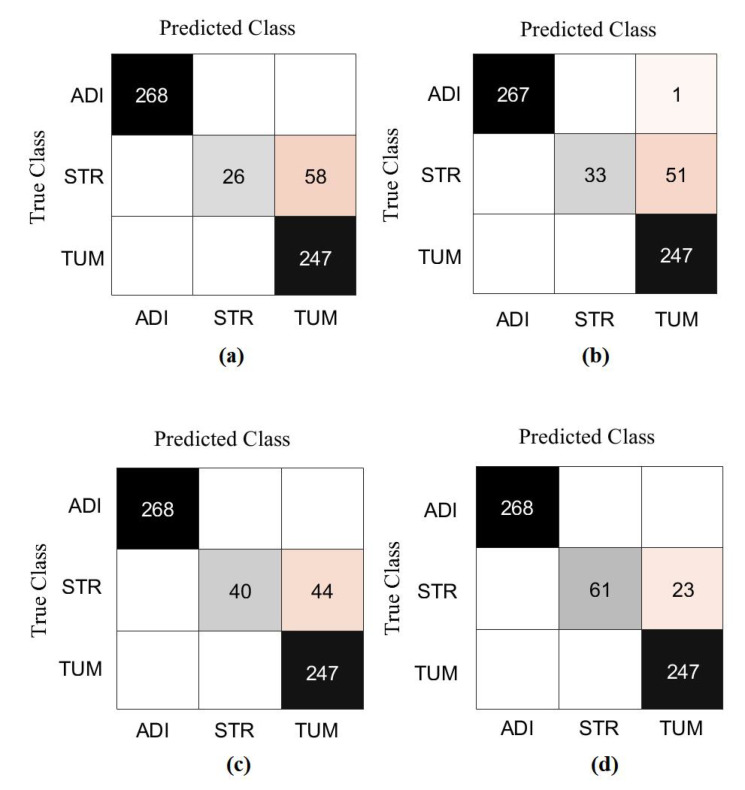
The confusion matrix results of CRC dataset obtained by: (**a**) ResNet-50 pre-trained network, (**b**) DeTraC, (**c**) *4S-DT*, and (**d**) *XDecompo*.

**Figure 9:**
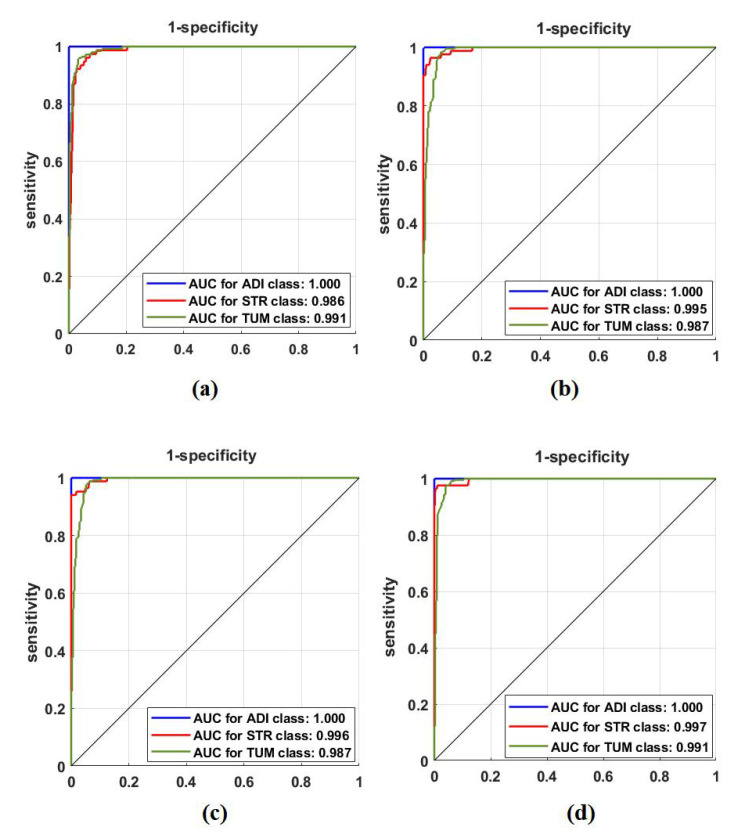
ROC analysis of the CRC test set obtained by: (**a**) ResNet-50 pre-trained network, (**b**) DeTraC, (**c**) *4S-DT*, and (**d**) *XDecompo*.

First, we used ResNet-50 on the 599 test images to investigate the performance of the proposed method, based on the fine-tuning strategy. ResNet-50 consists of 16 residual blocks with a depth of three layers, a 3 × 3 Max-Pooling layer, and a 1 × 1 Average-Pooling layer before the classification layer. Table 3 illustrates the adopted architecture used in our experiment. The model was trained with a learning rate of 0.0001 for CNN layers, 0.01 for FC layer, and the drop learning rate was set to 0.95 every 5 epochs with a minimum of 50 epochs and a mini-batch size of 50. The weight decay was set at 0.01 to avoid overfitting and 0.9 for the momentum. We also compared the performance of *XDecompo* with the *4s-DT*, *DeTraC*, and ResNet-50 ImageNet pre-trained network. For a fair comparison, we used the same parameter settings for each pre-trained model during the training process. The results obtained are summarised in Table 7. As demonstrated by Table 7, the best overall accuracy has been achieved by *XDecompo* with 97.44% and 90.87% for sensitivity and 97.82% for specificity, for classification test images into three classes compared to other models. Furthermore, Figure 8 shows the confusion matrix of the results obtained. Moreover, we compared the Area Under the receiver Curve (AUC) for each class between the true-positive rate (sensitivity) and false positive rate (1 specificity) obtained by ResNet-50 pre-trained network, *DeTraC*, *4S-DT* and *XDecompo*, see Figure 9. As shown in Figure 9, *XDecompo* has the highest AUC value in each class on the testing set of the CRC dataset. Figure 8 illustrates the confusion matrix obtained by each model for each class in the dataset.

### 4.5. Classification Performance on Brain Tumour Dataset

**Figure 10:**
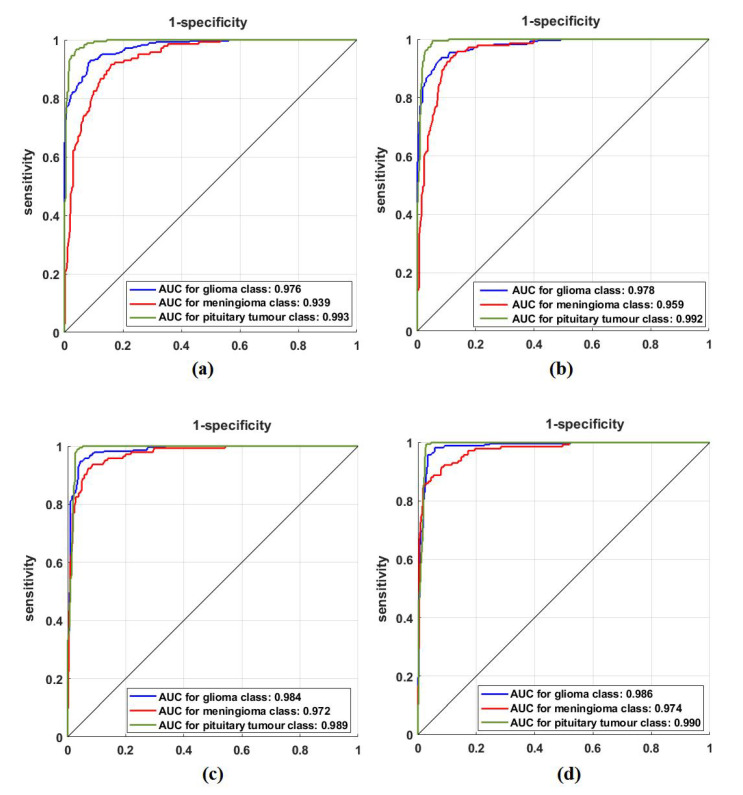
ROC analysis of the Brain tumour test set obtained by: (**a**) ResNet-50 pre-trained network, (**b**) DeTraC, (**c**) *4S-DT*, and (**d**) *XDecompo*.

**Figure 11:**
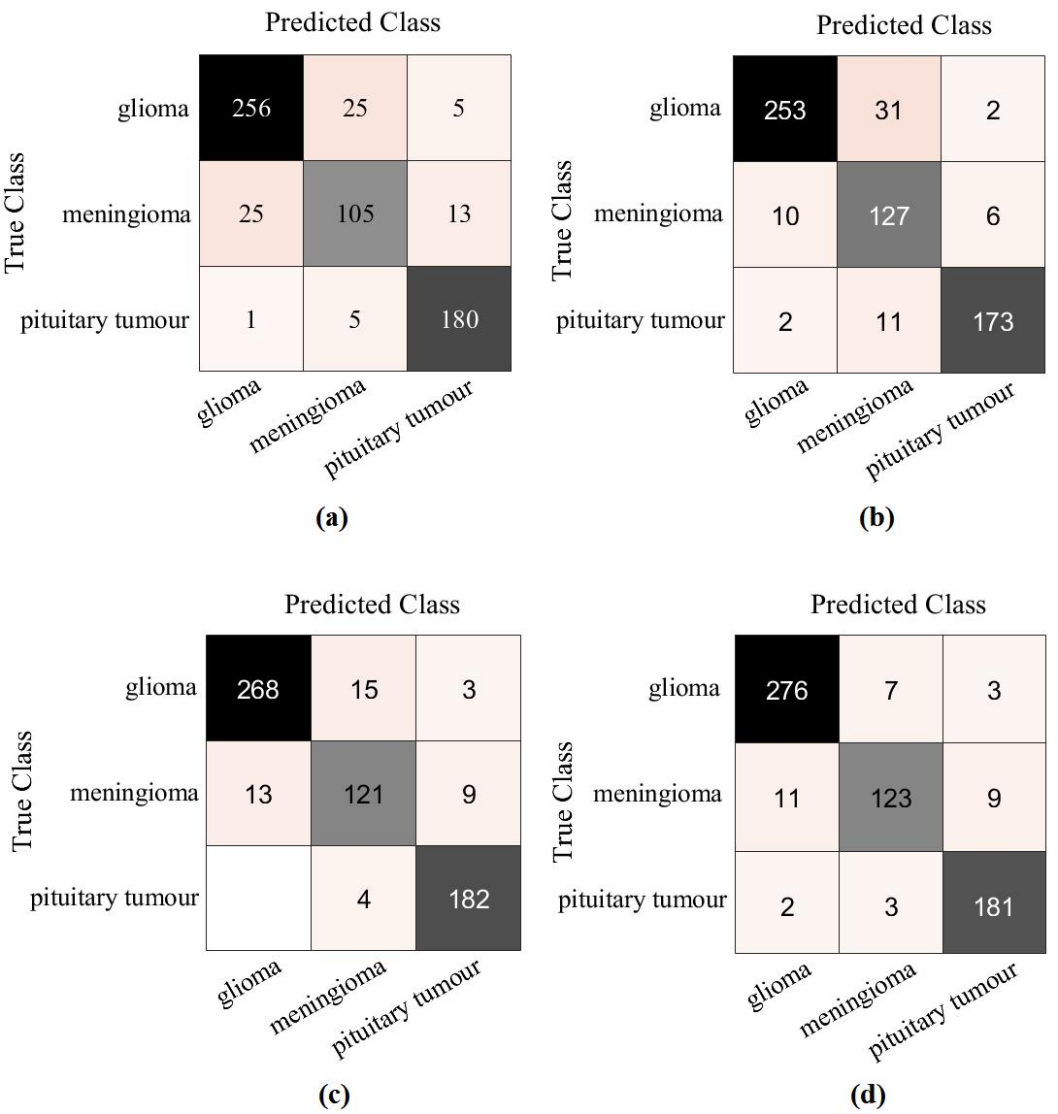
The confusion matrix results of Brain tumour dataset obtained by: (**a**) ResNet-50 pre-trained network, (**b**) DeTraC, (**c**) *4S-DT*, and (**d**) *XDecompo*.

For further investigation and to evidence the method’s generalisation and explainability, we evaluate the performance of *XDecompo* with ResNet-50 (where the fine-tuning mode was used) on the brain tumour test set, see Table 6. All images were resized to 224 × 224 pixels to be suitable for ResNet-50. *XDecompo* was trained for 50 epochs, 50 mini-batch sizes with a learning rate of 0.0001 for CNN layers, and 0.01 for FC layer. The drop learning rate schedule was set to 0.95 every 4 epochs, a value of 0.001 was set for the regularisation term, and 0.9 for the momentum. For a fair comparison, we used the same parameter settings for each pre-trained model during the training process. Table 8 shows the comparison results obtained by *XDecompo*, *4S-DT*, *DeTraC*, and the pre-trained network. Figure 10 shows the (AUC) for each class in the test set obtained by each model. As shown by Table 8, *XDecompo* has the highest overall accuracy with 96.21%, 93.28% for sensitivity, and 97.04% for specificity for classification test images into three classes compared to *4S-DT*, DeTraC, and the original classes based on the ResNet-50 pre-trained network. Figure 11 illustrates the confusion matrix obtained by each model for each class in the test set.

**Figure 12:**
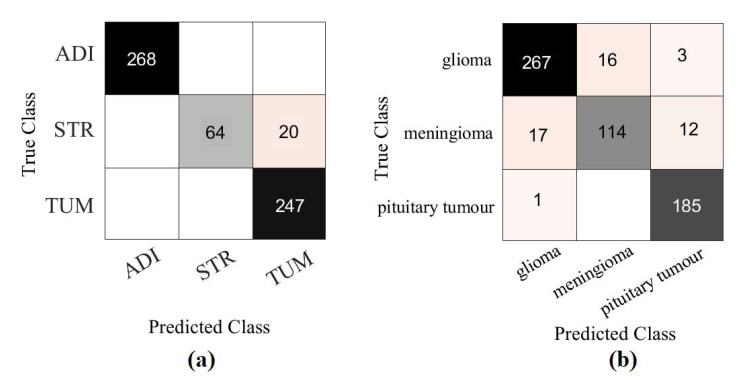
The confusion matrix results obtained in the test sets by using the AP-based class decomposition of *XDecompo* instead of the decomposition component of 4S-DT model: (**a**) CRC dataset, (**b**) Brain tumour dataset.

Moreover, we demonstrated the high quality of the decomposed classes of the AP-based class decomposition of *XDecompo*, where we used the same number of classes obtained by *XDecompo* in the downstream task as in the *4S-DT* model, see the third row in Table 5 and Table 6. The results obtained by *4S-DT* are reported in Table 9 to support and confirm the importance of the proposed class decomposition of the *XDecompo* model. Figure 12 illustrates the confusion matrix results obtained in the test sets using the AP-based class decomposition of *XDecompo* instead of the decomposition component of the 4S-DT model.

Having demonstrated experimentally and quantitatively the effectiveness of *XDecompo* on two datasets with variations in data irregularities, it is important to demonstrate that such a boosted performance is the result of the effective transferability of features. This can be best demonstrated visually to qualitatively assess the transferred features and their relationship to the predicted class.

Finally, we made a comparison between our results obtained by *XDecompo* model and different approaches that used the same labelled dataset for the classification of CRC and brain tumour datasets, see Table 10 and Table 11. Please note that the results were obtained by fine-tuning mode and only the weights of the last four layers were updated, see Table 3.

### 4.6. Feature Visualisation

**Figure 13:**
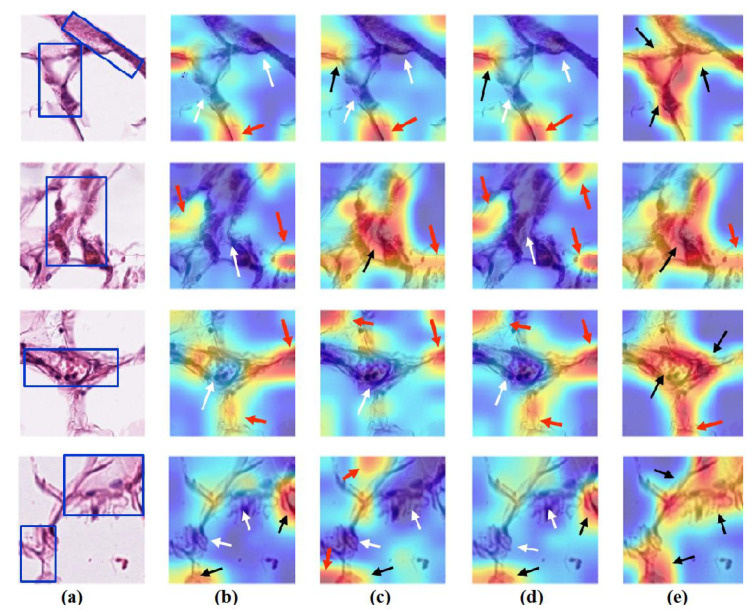
Visualisation of deep features for class ADI of CRC test set images obtained by each model: (**a**) original image with the region of interest covered in a blue rectangle, (**b**) ResNet-50 pre-trained network, (**c**) DeTraC, (**d**) *4S-DT*, and (**e**) *XDecompo*.

**Figure 14:**
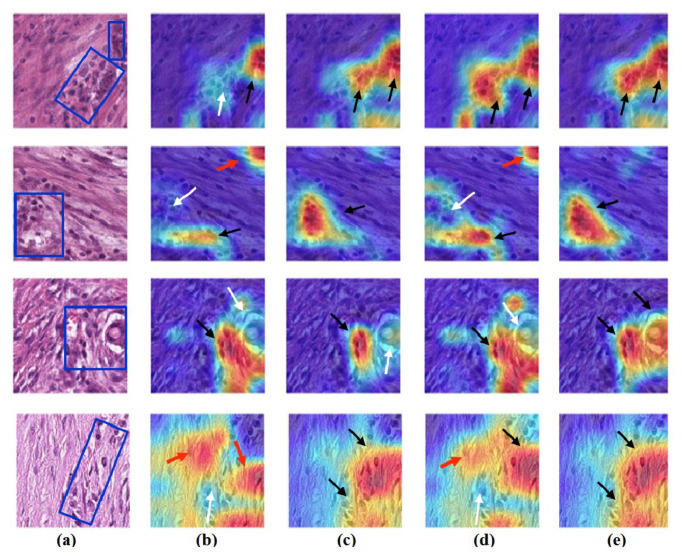
Visualisation of deep features for class STR of CRC test set images obtained by each model: (**a**) original image, (**b**) ResNet-50 pre-trained network, (**c**) DeTraC, (**d**) *4S-DT*, and (**e**) *XDecompo*.

**Figure 15:**
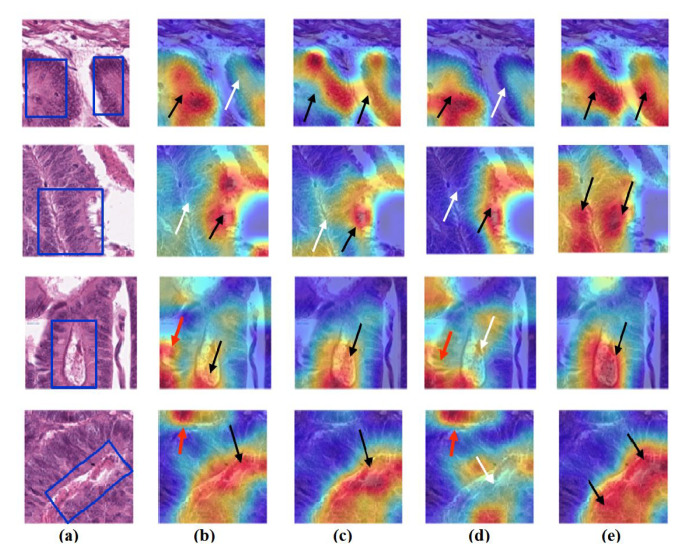
Visualisation of deep features for class TUM of CRC test set images obtained by each model: (**a**) original image, (**b**) ResNet-50 pre-trained network, (**c**) DeTraC, (**d**) *4S-DT*, and (**e**) *XDecompo*.

**Figure 16:**
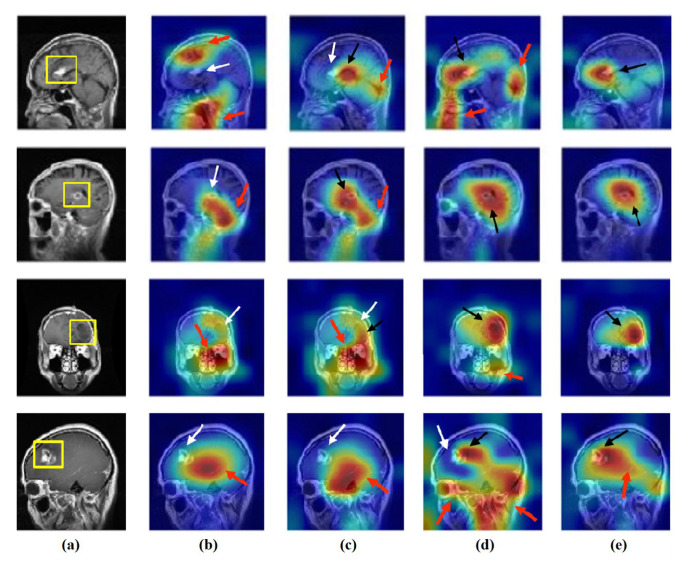
Visualisation of deep features for class glioma of brain tumour test set images obtained by each model: (**a**) original image, (**b**) ResNet-50 pre-trained network, (**c**) DeTraC, (**d**) *4S-DT*, and (**e**) *XDecompo*.

**Figure 17:**
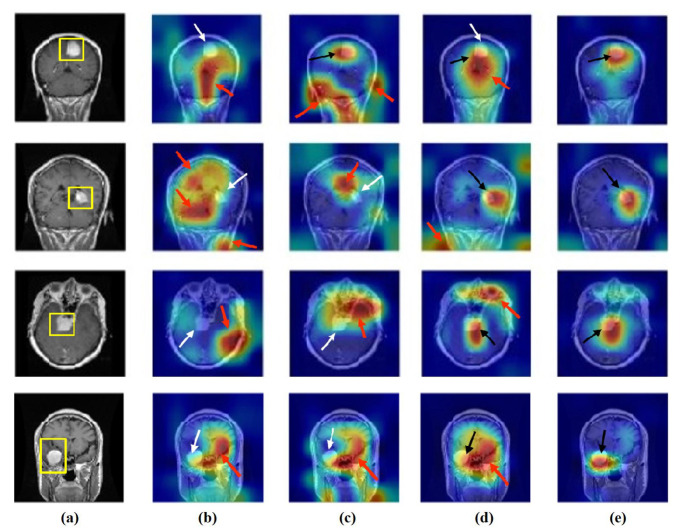
Visualisation of deep features for class meningioma of brain tumour test set images obtained by each model: (**a**) original image, (**b**) ResNet-50 pre-trained network, (**c**) DeTraC, (**d**) *4S-DT*, and (**e**) *XDecompo*.

**Figure 18:**
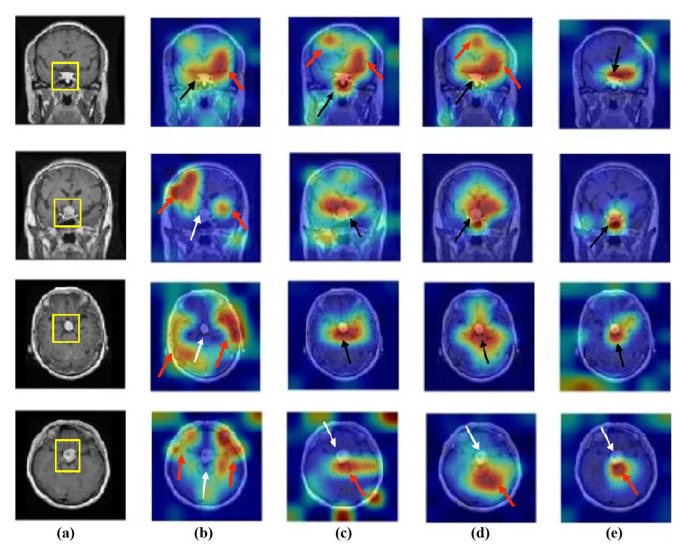
Visualisation of deep features for class pituitary tumours of brain tumour test set images obtained by each model: (**a**) original image, (**b**) ResNet-50 pre-trained network, (**c**) DeTraC, (**d**) *4S-DT*, and (**e**) *XDecompo*.

We generate the attention/heatmap of a post hoc explainable model for each model to better understand their behaviour. Here, we used Grad-CAM explainable model for each class in the CRC and brain tumour datasets, where the last convolutional layer was used to extract relevant rich features from the images and generate the final heatmap. In the heatmap, the areas where the CNN model has influenced to are highlighted in red and yellow colours, while the areas in blue colour are less related to the prediction. Figure 13, Figure 14 and Figure 15 show the heatmaps for ADI, STR, and TUM classes, respectively, in the test set of the CRC dataset for each model. Furthermore, Figure 16, Figure 17 and Figure 18 show the heatmaps for glioma, meningioma, and pituitary tumours, respectively, in the test set of the brain tumour dataset for each model. The black arrow refers to the correct region in which the model was able to attend during training, while the red and white arrows refer to the false misleading region and missing detection of the important region in the image, respectively.

As shown, *XDecompo* can localise more accurate pixels/regions for each class in the images compared *4S-DT* model, fine-tuned ResNet-50 network, and DeTraC. More precisely, in Figure 13, *XDecompo* was able to highlight all the adipose tissue regions (black arrows) compared with other models that failed to find all the adipose tissue regions (white arrows) and attended to other misleading regions (red arrows). Figure 14 and Figure 15 showed the ability of all models to find most of the stromal and epithelial cells, respectively. However, *XDecompo* outperformed other models in accurately detecting the whole epithelium in the images and avoiding misleading regions (red arrows). Similarly, in the brain tumour test set, *XDecompo* was able to attend accurately to the specific locations of the different tumour regions in all three classes, while other models attended to false regions (red arrows) and failed to accurately localise the whole tumour regions (white arrows). This experiment validates the highly accurate transferability capability of the proposed model and the high speciality of the features learnt by the model.

## 5. Discussion and Conclusions

Convolution neural network is one of the most successful approaches in deep learning and can be trained with various strategies. The transfer learning technique considers a practical solution to achieve a significant performance, especially in the medical image domain due to the limitation of the annotated samples. However, the irregularities in the dataset distribution remain the main challenge to providing a robust solution. In [19], *4S-DT* has demonstrated its effectiveness in coping with such challenging problems using a self-supervised mechanism (to generate pseudo-labels for a pretext task) with class decomposition method (to classify effectively samples in a downstream task). In this paper, we investigate the effectiveness and generalisation capabilities of a more robust version of *4S-DT*, which we call the *XDecompo* model in handling different medical image datasets. Unlike *4S-DT*, we used a self-supervised sample decomposition method with a convolutional autoencoder to generate the pseudo-labels in the pretext task. Moreover, in the downstream tasks, we proposed an automated class decomposition mechanism based on the affinity propagation approach. *XDecompo*’s class decomposition mechanism showed better performance (and hence a better transferability of the features) in comparison with *4S-DT*’s class decomposition. For the CRC dataset, the *XDecompo* model has achieved an overall accuracy of 97.44% with 90.87% for specificity and 97.82% for sensitivity to classify the test set into three classes. Furthermore, for the brain tumour dataset, *XDecompo* has achieved the highest overall accuracy of 96.21% with 93.28% for specificity and 97.04% for sensitivity. Qualitatively, the Grad-CAM map showed that *XDecompo* can accurately localise the most important textures (and regions of interest) in an input image, contributing to accurate predictions. To summarise, *XDecompo* achieves high classification accuracy (both quantitatively and qualitatively) in different medical image datasets based on fine-tuning mode, compared with the ResNet-50 pre-trained network, *DeTraC*, and *4S-DT* models.

## Figures and Tables

**Table 3:** Fine-tuned ResNet-50 architecture that we used in our experiments.

Layer Name	ResNet-50	Filter Size	Stride	Padding	# Filter
Conv5-1	res5c- branch2a	1 × 1 × 2048	1	0	512
Conv5-2	res5c- branch2b	3 × 3 × 512	1	1	512
Conv5-3	res5c- branch2c	1 × 1 × 512	1	0	2048
FC	Fully Connected	1 × 1	-	-	2048

**Table 4:** The classification performance of ResNet-50 pre-trained model on the pseudo-labelled Colorectal cancer and Brain tumour datasets.

AE Model	Dataset	(Eps)	# Labels	ACC (%)
SAE	Colorectal cancer	4.5	8	75.80
	Brain tumour	4.2	6	79.12
CAE	Colorectal cancer	4	4	87.36
	Brain tumour	2	3	92.79

**Table 5:** The number of instances in original classes and after class decomposition of the colorectal cancer dataset using k-means and AP clustering algorithms.

k-means clustering	Dataset A	ADI				STR		TUM		
	# instances	1070				337		986		
	Dataset B		ADI_1	ADI_2		STR_1	STR_2		TUM_1	TUM_2
	# instances		666	404		171	166		406	580
AP clustering	Dataset B	ADI_1	ADI_2	ADI_3	ADI_4	STR_1	STR_2	TUM_1	TUM_2	TUM_3
	# instances	377	270	222	201	171	166	381	371	234
	data set B	ADI_1	ADI_2	ADI_3	ADI_4	STR_1	STR_2	TUM_1	TUM_2	TUM_3
	# instances	420	374	127	149	171	166	189	394	403

**Table 6:** The number of instances in original classes and after class decomposition of the brain tumour dataset using k-means and AP clustering algorithms.

k-means clustering	Dataset A	glioma			meningioma		pituitary tumour		
	# instances	1140			565		744		
	data set B	GLI_1	GLI_2		MEN_1	MEN_2		PIT_1	PIT_2
	# instances	577	563		298	267		426	318
AP clustering	Dataset B	GLI-1	GLI-2	GLI-3	MEN-1	MEN-2	PIT-1	PIT-2	PIT-3
	# instances	455	529	156	290	275	214	322	208
	Dataset B	GLI-1	GLI-2	GLI-3	MEN-1	MEN-2	PIT-1	PIT-2	PIT-3
	# instances	547	308	285	298	267	311	313	120

**Table 7:** Overall classification performance of each model on testing set of the CRC dataset.

Layer	ResNet-50 Pre-Trained			DeTraC			*4S-DT*			*XDecompo*		
	ACC	SN	SP	ACC	SN	SP	ACC	SN	SP	ACC	SN	SP
	(%)	(%)	(%)	(%)	(%)	(%)	(%)	(%)	(%)	(%)	(%)	(%)
FC	90.81	78.17	94.79	91.48	80.34	95.17	92.82	82.93	95.92	95.49	89.28	97.44
Conv5-3	90.65	77.67	94.69	92.15	81.34	95.54	92.32	81.74	95.64	94.82	87.97	97.06
Conv5-2	90.65	77.67	94.69	91.65	80.51	95.26	92.98	83.33	96.02	94.99	88.36	97.15
Conv5-1	90.31	76.98	94.50	91.31	79.63	95.07	92.65	82.54	95.83	96.16	90.87	97.82

**Table 8:** Overall classification performance of each model on the testing set of the Brain tumour dataset.

Layer	ResNet-50 Pre-Trained			DeTraC			*4S-DT*			*XDecompo*		
	ACC	SN	SP	ACC	SN	SP	ACC	SN	SP	ACC	SN	SP
	(%)	(%)	(%)	(%)	(%)	(%)	(%)	(%)	(%)	(%)	(%)	(%)
FC	87.31	86.16	93.74	89.91	88.84	94.97	91.70	90.95	95.82	92.52	91.62	96.15
Conv5-3	86.29	87.01	93.75	90.24	90.21	95.36	92.52	91.99	96.32	92.19	91.71	96.03
Conv5-2	86.67	86.71	93.62	89.26	89.66	95.01	93.00	92.12	96.43	92.84	91.52	96.15
Conv5-1	87.96	86.57	93.84	89.91	90.14	95.19	92.84	92.05	96.40	94.30	93.27	97.04

**Table 9:** Overall classification performance of the *4S-DT* model on the CRC and brain tumour testing sets.

Layer	CRC Dataset			Brain Tumour Dataset		
	ACC	SN	SP	ACC	SN	SP
	(%)	(%)	(%)	(%)	(%)	(%)
FC	95.49	89.28	97.44	87.31	83.50	93.20
Conv5-3	96.49	92.47	98.04	88.45	85.61	93.91
Conv5-2	96.66	92.06	98.10	89.10	86.24	94.42
Conv5-1	96.68	92.06	98.20	92.03	90.84	95.88

**Table 10:** Comparison between the result obtained by *XDecompo* and different approaches that used the same labelled dataset for the classification of CRC images.

Ref.	Method	ACC (%)
[64]	Multitask ResNet-50	95.0
[64]	CNN-ResNet-50	93.60
[65]	Ensemble DNN	92.83
[66]	CNN-Xception	94.4
[67]	CNN-VGG19	94.3
	*XDecompo*	96.16

**Table 11:** Comparison between the result obtained by *XDecompo* and different approaches that used the same labelled dataset for the classification of brain tumour images.

Ref.	Method	ACC (%)
[68]	7-layered CNN	84.19
[69]	BoW + SVM	91.28
[70]	CNN + KELM	93.68
[71]	CNN-transfer learning	92.00
[72]	CapsNet	90.89
	*XDecompo*	94.30

## Data Availability

We have used publicly available datasets. For the colorectal cancer histology dataset, we used the labelled and unlabelled datasets from [58]. For the brain tumour dataset, the unlabelled dataset is publicly available for download at (https://www.kaggle.com/datasets/navoneel/brain-mri-images-for-brain-tumor-detection accessed on 14 November 2022), and the labelled dataset is available at [59].
